# Risk of gastrointestinal intolerance and complications associated with homemade versus commercial enteral nutrition in critically ill patients: a single-center retrospective cohort study

**DOI:** 10.3389/fnut.2026.1803903

**Published:** 2026-04-23

**Authors:** Yingying Wang, Quanquan He, Ming Xia, Weiwei Ni, Shichao Zhu

**Affiliations:** 1 Intensive Care Unit, Intensive Care Key Laboratory of Zhengzhou City, Henan Provincial People's Hospital, People's Hospital of Zhengzhou University, Zhengzhou, China; 2 School of Nursing, Shandong Second Medical University, Weifang, China; 3 Department of Nursing, Affiliated Hospital of Shandong Second Medical University, Weifang, China

**Keywords:** diarrhea, enteral nutrition, intensive care unit, retrospective cohort study, tolerance

## Abstract

**Background:**

Critically ill ICU patients often fail to achieve target caloric intake due to gastrointestinal intolerance. Although commercial enteral nutrition (CEN) formulas are the standard of care, emerging evidence suggests they may worsen intolerance in certain susceptible populations. Individualized commercial formulation (ICF) may help reduce related complications, but its safety and effectiveness compared to commercial formulas remain insufficiently validated.

**Objective:**

To compare the associations of CEN, mixed feeding, and ICF with gastrointestinal tolerance and complication risk in ICU patients.

**Methods:**

In this single-center retrospective cohort study, 605 adult ICU patients receiving tube-fed EN at Henan Provincial People’s Hospital between January 2023 and January 2025 were included. Patients were classified into Commercial (*n* = 477), Mixed (*n* = 84), and ICF (*n* = 44) groups according to EN source. The primary outcome was diarrhea. Secondary outcomes included feeding interruption, gastric residual volume, constipation, hyperglycemia, and composite gastrointestinal complications. Multivariable logistic regression was used to adjust for baseline and feeding-related factors. Missing data were handled by multiple imputation. Propensity score matching and APACHE II–stratified analyses were performed as sensitivity analyses.

**Results:**

Compared with the Commercial group, the ICF group had a lower incidence of diarrhea (13.6% vs. 41.5%; aOR = 0.27, 95% CI 0.11–0.68; *p* = 0.005), fewer feeding interruptions (IRR = 0.29, 95% CI 0.18–0.45; *p* < 0.001), higher mean daily caloric intake (951 ± 218 vs. 893 ± 272 kcal/day, *p* < 0.001), and lower gastric residual volume (median 0 vs. 0–150 mL; *p* < 0.001). Hyperglycemia was also less frequent in the ICF group (25.0% vs. 99.6%, aOR < 0.001), although the overall incidence was high under the study definition. The risk of composite gastrointestinal complications was reduced by approximately 64% (aOR = 0.36, 95% CI 0.19–0.70; *p* = 0.002). No significant differences were observed in the Mixed group. Results were consistent in sensitivity analyses.

**Conclusion:**

In critically ill ICU patients, ICF was associated with improved gastrointestinal tolerance and lower risks of diarrhea and selected complications compared with CEN. However, these findings should be interpreted cautiously given the observational design.

## 1 Introduction

Enteral nutrition (EN) is a cornerstone of supportive therapy for critically ill patients in the ICU. Compared with parenteral nutrition, EN confers substantial physiological advantages, including preservation of gut barrier integrity, modulation of the intestinal microbiota, and a reduced risk of infectious complications (1–3). Accordingly, the most recent guidelines from both the European Society for Clinical Nutrition and Metabolism (ESPEN) and the American Society for Parenteral and Enteral Nutrition (ASPEN) strongly recommend the early initiation of EN in hemodynamically stable critically ill patients, with the aim of attenuating catabolism, improving clinical outcomes, and potentially shortening ICU length of stay (1, 2). However, gastrointestinal intolerance remains a major challenge in routine clinical practice. Manifestations such as diarrhea, increased gastric residual volume, constipation, and feeding interruption are frequently encountered, with reported incidences ranging from 30.5 to 65.7%. These complications are closely associated with inadequate caloric delivery, prolonged duration of mechanical ventilation, and increased mortality (4). Currently, commercially manufactured EN formulas are widely used in clinical practice. These industrially standardized products offer well-defined macro-and micronutrient compositions, high microbiological safety, stable energy density, and ease of administration (5, 6). In accordance with guideline recommendations, a variety of formulations are available to meet different metabolic requirements, including high–energy density formulas, peptide-based pre-digested formulas, and diabetes-specific formulations (1, 7). Despite these advantages, an increasing body of evidence suggests that CEN formulas may exacerbate gastrointestinal intolerance in certain vulnerable patient populations (8). Proposed mechanisms include high osmolarity, the presence of emulsifiers and stabilizers, and the relative lack of natural dietary fiber, which may impair colonic short-chain fatty acid production and adversely affect intestinal function (9–11).

In contrast, ICF prepared either from pureed whole foods or through hospital-controlled compounding of medical-grade nutritional powders may provide a more physiologically congruent mode of nutrient delivery (12, 13). Potential advantages include preservation of the natural food matrix, a more diverse and adaptable fiber composition, and the presence of bioactive compounds (12, 13). Collectively, these characteristics may enhance intestinal motility, support microbial diversity, and promote colonic water absorption (12, 13). Several observational studies and small randomized trials have reported that blenderized or natural-food–based enteral feeding significantly reduces the incidence of diarrhea and improves feeding tolerance in specific populations, such as patients with neurological disorders or those undergoing cardiac surgery (14, 15). These benefits have been attributed, at least in part, to lower osmotic load and the maintenance of more intact food structures. Nevertheless, existing evidence remains inconsistent. Some studies have raised concerns regarding the potential risks of non-standardized homemade enteral feeding, including microbial contamination, variability in nutrient composition, and feeding tube occlusion. These safety concerns underscore the need for rigorous evaluation of ICF under controlled preparation conditions (16).

Although ICF has emerged as a pragmatic and potentially advantageous alternative, its safety and effectiveness relative to commercial formulas have not been adequately validated in large-scale, systematic studies (14, 15). Several critical gaps persist in the current literature. Most available studies are limited by small sample sizes and substantial heterogeneity in the definitions of gastrointestinal intolerance. Moreover, insufficient adjustment for key confounding factors such as disease severity and concomitant medications, particularly antibiotics that may disrupt the gut microbiota and increase the risk of diarrhea further limits the interpretability of existing findings (17–19). In addition, comprehensive assessments of feeding continuity, metabolic complications, and composite gastrointestinal outcomes remain scarce. These limitations collectively hinder the accumulation of high-quality evidence and impede the refinement of clinical practice guidelines for nutritional support in critically ill patients (20–22).

To address these knowledge gaps, we conducted a single-center retrospective cohort study involving 605 ICU patients to compare the effects of commercial, mixed, and ICF on gastrointestinal tolerance and complication risk. We hypothesized that, when prepared under strict hospital-controlled conditions, ICF would be independently associated with improved feeding tolerance and a lower risk of diarrhea compared with commercial formulas. By employing multivariable regression analyses and a series of sensitivity analyses, this study aims to provide a more robust evidence base for nutritional support strategies in critically ill patients and to inform the design of future large-scale randomized controlled trials.

## 2 Methods

### 2.1 Study design

This study was a single-center retrospective cohort study designed to evaluate the associations between different EN sources and gastrointestinal tolerance as well as clinical outcomes in critically ill patients. The study was conducted in the ICU of Henan Provincial People’s Hospital between January 2023 and January 2025. The protocol was approved by the hospital’s ethics review committee (Approval No. 2022-55) and was conducted in accordance with the principles of the Declaration of Helsinki.

### 2.2 Study population and grouping

Eligible participants met the following inclusion criteria: (1) age ≥18 years; and (2) receipt of tube-fed EN during ICU hospitalization. Exclusion criteria were: (1) age <18 years; (2) no EN administered during the ICU stay or exclusive receipt of parenteral nutrition; and (3) missing EN implementation records or inability to ascertain outcome measures. A total of 605 consecutive patients were ultimately included. Patients were categorized according to the source of EN received during the observation period into three groups: Commercial group (*n* = 477), Mixed group (*n* = 84), and ICF group (*n* = 44). Group assignment was determined based on the type(s) of EN administered during the study window.

#### 2.2.1 Commercial group

Patients in the Commercial group received only industrially manufactured, standardized EN formulas. Depending on patients’ clinical conditions (e.g., metabolic requirements or comorbidities), selected products included standard whole-protein formulas (e.g., Nutrison® 1.0 kcal/mL), high–energy density formulas (e.g., Nutrison® Energy 1.5 kcal/mL), peptide-based pre-digested formulas (e.g., Peptisorb®), and diabetes-appropriate formulas (e.g., Nutrison Advanced Diason®). These formulas have fixed macro- and micronutrient compositions and align with ESPEN guideline recommendations.

#### 2.2.2 ICF group

Patients in the ICF group received hospital-compounded clinical EN formulations prepared under the supervision of the hospital nutrition department (Hospital-Compounded Clinical Formulation). Importantly, this approach differs from non-standardized household blenderized feeding. In this study, ICF was reconstituted from standardized medical nutritional powders tailored to specific metabolic needs (e.g., electrolyte disturbances or fluid restriction). Standard regimens included low-potassium or high-sodium base powders combined with whey protein powder at prespecified ratios (for example, a standard formulation comprised 66 g of base powder plus 4 g of whey protein powder dissolved in 250 mL of warm water, yielding an energy density of approximately 1.0 kcal/mL). To ensure microbiological safety and dosing accuracy, all formulations were prepared in a clean environment within the hospital nutrition compounding room in strict accordance with national food safety standards; finished products were dispensed as single-dose portions, and labels explicitly specified a 24-h validity period and detailed pump-infusion instructions.

#### 2.2.3 Mixed group

The Mixed group comprised patients who, due to changes in clinical condition during the observation period, received both CEN and ICF either sequentially or concurrently. In this retrospective dataset, detailed information on the relative proportion and duration of each feeding modality was not consistently available; therefore, exposure was classified qualitatively rather than quantitatively.

In routine clinical practice, enteral nutrition was generally initiated within 24–72 h after ICU admission in accordance with guideline recommendations. CEN formulas were used as the first-line nutritional strategy for all eligible patients. The decision to initiate ICF were not predefined but was made following multidisciplinary evaluation by ICU physicians and clinical dietitians. ICF was typically introduced when patients exhibited poor tolerance to standard commercial formulas or when commercial products were considered insufficient to meet individualized metabolic or electrolyte requirements. To improve transparency regarding nutritional comparability, we summarized the typical macronutrient composition and available information on dietary fiber content of representative enteral formulations used in each group based on product specifications and hospital compounding protocols (Supplementary Table S1). Although commercial formulas had standardized nutrient profiles, ICF compositions were individualized according to patients’ metabolic and electrolyte requirements, which may have introduced variability in micronutrient and fiber content.

### 2.3 Data collection and quality control

All data were extracted from the hospital electronic medical record system (HIS), including baseline characteristics, clinical outcomes, detailed information on EN implementation, and daily laboratory indices. However, detailed information on certain treatment variables, including corticosteroid use, insulin therapy, and precise macronutrient composition of enteral formulations, was not consistently available in the retrospective dataset. To minimize ascertainment bias, data extraction was independently performed by two investigators who were blinded to the study hypothesis; discrepancies were adjudicated by a senior investigator. All data were anonymized to protect patient privacy. Although nutritional risk screening using the NRS-2002 tool was routinely performed in clinical practice, these data were not available in the retrospective dataset and therefore could not be included in the present analysis.

### 2.4 Outcome definitions

The primary outcome was the incidence of diarrhea. Diarrhea was defined in accordance with the World Health Organization (WHO) criteria and the validated ICU nutrition research protocol described by Dionne et al. (23): passage of loose or watery stools (Bristol Stool Form Scale types 6–7) at least three times within 24 h (23–25). For patients who developed diarrhea, the time of onset, daily stool frequency, and duration were recorded to quantify diarrhea burden.

Secondary outcomes captured gastrointestinal tolerance, feeding continuity, and metabolic complications. Gastrointestinal tolerance was primarily assessed using gastric residual volume (GRV) (26). Gastric retention was defined as a single-day GRV > 100 mL or a clinician-documented retention event requiring intervention (27), This threshold is more stringent than the ASPEN-recommended range (250–500 mL) and was applied in our clinical practice to facilitate early identification of feeding intolerance. Although the 2016 ASPEN guideline suggests a more permissive threshold for holding feeds (250–500 mL), this study applied a stricter monitoring threshold (>100 mL) to facilitate early identification of intolerance signals (1). Additional secondary outcomes included enteral feeding interruption (defined as any temporary cessation of infusion), assessed by incidence, frequency, and cumulative duration (28); hyperglycemia (blood glucose >10 mmol/L) (29), Hyperglycemia was defined as any blood glucose measurement >10 mmol/L during ICU stay. Given the high frequency of glucose monitoring in critically ill patients, this definition was intended to maximize sensitivity but may capture transient or clinically mild elevations; constipation (no bowel movement for ≥3 consecutive days) (30); and a composite gastrointestinal complication outcome defined as the occurrence of any gastrointestinal complication (31).

### 2.5 Statistical analysis

Because this was a retrospective analysis, sample size was determined by the number of eligible cases available during the study period. Although the ICF group was relatively small (*n* = 44), based on preliminary assumptions (diarrhea incidence ~40% in the Commercial group vs. ~15% in the ICF group), a post-hoc power analysis indicated that the study had >80% power (two-sided *α* = 0.05) to detect between-group differences.

All analyses were two-sided, with *p* < 0.05 indicating statistical significance. Secondary outcomes were considered exploratory, and no formal adjustment for multiple comparisons was applied. Analyses were conducted using R (version 4.3.0) and SPSS (version 26.0). Normality of continuous variables was assessed using the Shapiro–Wilk test. Normally distributed variables are presented as mean ± standard deviation, non-normally distributed variables as median [interquartile range, IQR], and categorical variables as n (%). Between-group comparisons across the three groups were performed using ANOVA or the Kruskal–Wallis test for continuous variables and the χ^2^ test or Fisher’s exact test for categorical variables; Bonferroni correction was applied for *post hoc* pairwise comparisons following ANOVA. Diarrhea incidence was compared using the χ^2^ test. Kaplan–Meier curves with the log-rank test were used to evaluate the probability of remaining diarrhea-free.

The number of feeding interruptions was analyzed using Poisson regression, with results reported as IRRs with 95% confidence intervals (CIs). Binary outcomes were analyzed using multivariable logistic regression, and continuous outcomes using multivariable linear regression. Given the relatively small sample size of the ICF group, we additionally evaluated model stability by calculating the events-per-variable (EPV) ratio for each multivariable model. Sensitivity analyses using reduced-variable models were also performed to assess the robustness of the findings. Models were constructed sequentially, including an unadjusted model, a model adjusted for baseline characteristics, and a fully adjusted model additionally accounting for clinical interventions and feeding-related factors. Results are reported as aOR or adjusted regression coefficients (*β*) with 95% CIs, as appropriate. Missing data were handled using multiple imputation with chained equations (MICE). Sensitivity analyses included: (1) a two-group comparison excluding the Mixed group; (2) 1:1 propensity score matching to balance baseline covariates; and (3) APACHE II–stratified analyses with interaction testing. Data and code are available upon reasonable request in accordance with ICMJE policies.

### 2.6 Ethics statement

This study was conducted in strict accordance with ethical requirements and was approved by the Ethics Review Committee of Henan Provincial People’s Hospital (Approval No. 2022-55). Given the retrospective design and anonymization of patient data, the requirement for informed consent was waived.

## 3 Results

### 3.1 Baseline characteristics of the study population

A total of 605 critically ill patients were included in the study (Commercial group, *n* = 477; Mixed group, *n* = 84; ICF group, *n* = 44). The baseline demographic and clinical characteristics are summarized in Table 1. The three groups were well-balanced regarding age, gender, disease severity, and the distribution of primary diagnoses (*p* > 0.05 for all comparisons). Similarly, no significant differences were observed in the use of antibiotics or sedatives. However, the utilization of vasoactive drugs differed significantly among the groups (*p* = 0.003), with the highest usage rate in the Commercial group (64.6%) and the lowest in the ICF group (43.2%). Consequently, this variable was identified as a potential confounder and was adjusted for in all subsequent multivariable analyses.

**Table 1:** Baseline characteristics and diagnosis distribution.

Variables	Commercial group (*n* = 477)	Mixed group (*n* = 84)	ICF group (*n* = 44)	*p-*value
Demographics and scores				
Age (years), median [IQR]	57.0 [45.0–70.0]	58.0 [41.8–70.0]	52.5 [46.5–64.0]	0.500
Male sex, *n* (%)	295 (62.0%)	55 (65.5%)	28 (63.6%)	0.820
APACHE II score, median [IQR]	18.0 [15.0–23.0]	17.0 [15.0–22.0]	16.5 [14.0–25.0]	0.397
Admission GCS/RASS, median [IQR]	3.0 [−1.0–8.0]	2.5 [−1.0–6.0]	5.0 [2.8–9.0]	0.052
Medications, *n* (%)				
Antibiotics	465 (97.5%)	83 (98.8%)	44 (100.0%)	0.441
Sedatives	424 (88.9%)	75 (89.3%)	35 (79.5%)	0.174
Vasoactive drugs	308 (64.6%)	43 (51.2%)	19 (43.2%)	0.003
Primary diagnosis, *n* (%)				0.939
Neurological disease	221 (46.3%)	34 (40.5%)	21 (47.7%)	
Respiratory disease	104 (21.8%)	22 (26.2%)	10 (22.7%)	
Cardiovascular disease	38 (8.0%)	9 (10.7%)	2 (4.5%)	
Trauma/surgical	28 (5.9%)	5 (6.0%)	3 (6.8%)	
Gastrointestinal disease	21 (4.4%)	6 (7.1%)	3 (6.8%)	
Sepsis/infection	10 (2.1%)	2 (2.4%)	1 (2.3%)	
Other	55 (11.5%)	6 (7.1%)	4 (9.1%)	

Data are presented as *n* (%) for categorical variables or median [interquartile range, IQR] for continuous variables; continuous variables were compared using the Kruskal-Wallis test; categorical variables were compared using the Chi-square test; APACHE II, acute physiology and chronic health evaluation II; GCS, Glasgow Coma Scale; RASS, Richmond Agitation-Sedation Scale.

### 3.2 EN implementation and interruptions

Details of EN implementation are presented in Table 2. Despite a shorter median duration of EN compared to the Mixed group, the ICF group achieved the highest mean daily caloric intake (951 ± 218 kcal/day), significantly exceeding that of the Commercial and Mixed groups (*p* < 0.001). This was achieved through a higher infusion volume, compensating for the lower caloric density of the homemade formula. Notably, the ICF group demonstrated superior feeding continuity, characterized by the lowest incidence (29.5%) and frequency of interruptions. Poisson regression analysis confirmed that the use of ICF was independently associated with a 71% reduction in the rate of interruptions compared to the Commercial group (Incidence Rate Ratio = 0.29, 95% CI: 0.18–0.45; *p* < 0.001).

**Table 2:** EN Implementation characteristics.

Variables	Commercial group (*n* = 477)	Mixed group (*n* = 84)	ICF group (*n* = 44)	*p*-value
Nutritional intake				
Duration of EN (days), median [IQR]	11.0 [8.0–16.7]	14.0 [8.0–22.3]	8.0 [6.0–13.0]	< 0.001*
Daily caloric intake (kcal/d), mean ± SD	893 ± 272	655 ± 145	951 ± 218	< 0.001^#^
Daily volume (mL/d), mean ± SD	878 ± 206	1,170 ± 260	1,087 ± 249	< 0.001^#^
Infusion rate (mL/h), mean ± SD	46.4 ± 62.4	33.3 ± 9.9	33.1 ± 10.7	0.060^#^
Route and history, *n* (%)				
Gastric tube feeding	431 (90.4%)	63 (75.0%)	34 (77.3%)	< 0.001^Δ^
Nasointestinal tube feeding	123 (25.8%)	22 (26.2%)	10 (22.7%)	0.898*^Δ^*
History of fasting	340 (71.3%)	13 (15.5%)	4 (9.1%)	< 0.001^Δ^
Tolerance (Interruptions)				
Incidence of interruption, n (%)	323 (67.7%)	42 (50.0%)	13 (29.5%)	< 0.001^Δ^
Frequency (times/patient), mean ± SD	1.54 ± 1.54	0.82 ± 1.01	0.44 ± 0.73	< 0.001^#^
Duration of interruption (h), median [IQR]	0.3 [0.0–1.0]	12.0 [0.0–48.0]	0.0 [0.0–24.0]	0.007*

*Indicates Kruskal-Wallis test; Δ indicates Chi-square test; # indicates ANOVA.

### 3.3 Incidence of diarrhea and multivariable analysis

**Figure 1:**
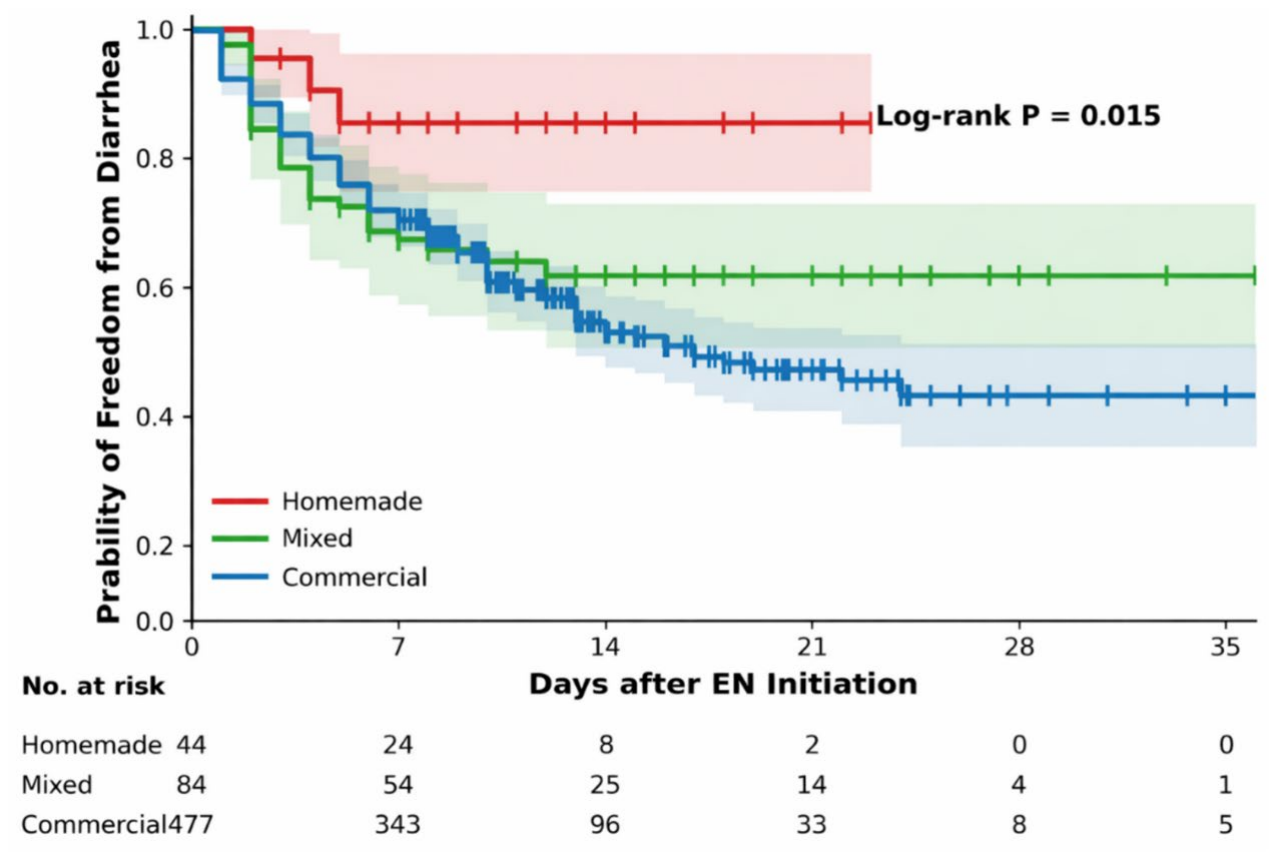
Kaplan–meier estimates of freedom from diarrhea. Survival curves show the probability of remaining diarrhea-free for the homemade (red), mixed (green), and commercial (blue) groups. Shaded areas represent 95% CI. Patients in the ICF group demonstrated a significantly higher probability of freedom from diarrhea compared with the commercial group (Log-rank *p* = 0.015).

Significantly lower in the ICF group (13.6%) compared to the Commercial (41.5%) and Mixed groups (35.7%; *p* = 0.001) (Table 3). Among patients who developed diarrhea, those in the ICF group experienced milder symptoms, indicated by a lower daily stool frequency. Kaplan–Meier survival analysis further demonstrated that patients in the ICF group had a significantly higher probability of remaining diarrhea-free throughout the treatment period (Log-rank *p* = 0.015) (Figure 1).

**Table 3:** Diarrhea characteristics.

Variables	Commercial group (*n* = 477)	Mixed group (*n* = 84)	ICF group (*n* = 44)	*p*-value
Incidence				
Diarrhea occurrence, *n* (%)	198 (41.5%)	30 (35.7%)	6 (13.6%)	0.001
Characteristics of events	(*n* = 198)	(*n* = 30)	(*n* = 6)	
Time to onset (days), median [IQR]	5.0 [2.0–8.0]	3.0 [2.0–4.8]	4.0 [2.5–4.8]	0.121
Daily stool frequency (times/day), mean ± SD	5.49 ± 1.52	4.93 ± 1.70	3.83 ± 1.17	0.001
Total duration of diarrhea (days), median [IQR]	2.0 [1.0–3.0]	2.0 [2.0–4.0]	2.0 [1.2–2.8]	0.054

Differences in incidence were analyzed using the Chi-square test. Characteristics of diarrhea events (onset, frequency, duration) were compared among patients who developed diarrhea using ANOVA or the Kruskal-Wallis test.

**Figure 2:**
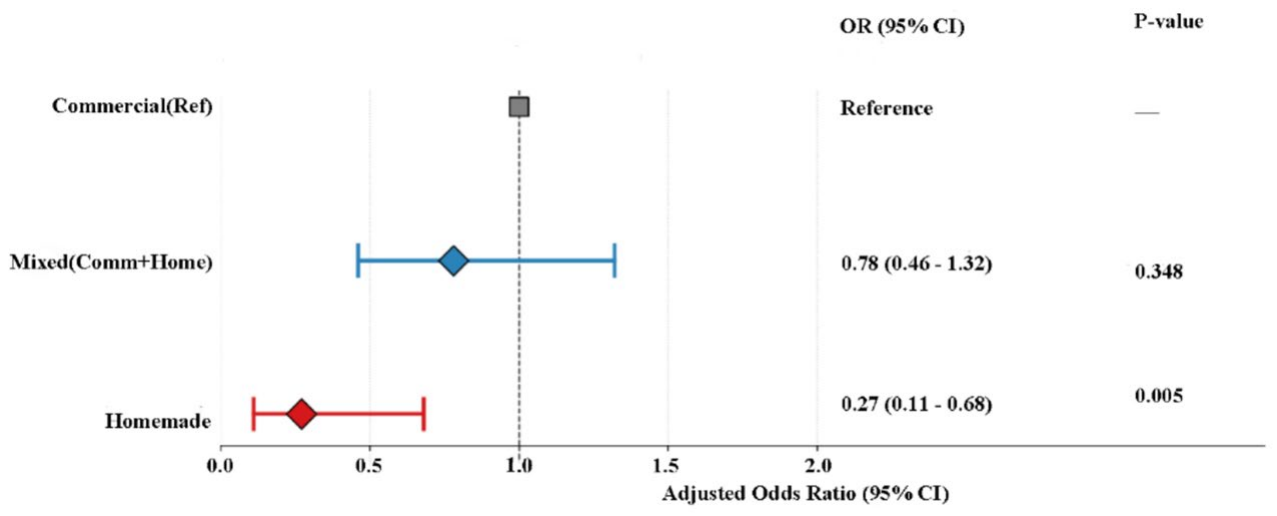
Adjusted odds ratios for diarrhea risk (Model 3). The plot shows aOR and 95% CI from the fully adjusted logistic regression model. The commercial group serves as the reference. ICF (red) was independently associated with a significant reduction in diarrhea risk (*p* = 0.005), whereas the mixed group (green) showed no significant difference.

Multivariable logistic regression analysis confirmed the robustness of this protective effect (Table 4). For the primary outcome, the EPV was approximately XX, indicating that model stability may be modestly limited; however, sensitivity analyses using reduced-variable models yielded consistent effect estimates. After adjusting for baseline characteristics and clinical interventions (including antibiotics and vasoactive drugs), patients receiving ICF had a 73% lower risk of developing diarrhea compared to the Commercial group (aOR = 0.27, 95% CI: 0.11–0.68; *p* = 0.005) (Figure 2). No statistically significant reduction in diarrhea risk was observed in the Mixed group.

**Table 4:** Multivariable logistic regression analysis for the risk of Diarrhea.

Comparison	Model 1 (Crude) OR (95% CI)	*p*-value	Model 2 (Adjusted)^1^ aOR (95% CI)	*p-*value	Model 3 (Fully adjusted)^2^ aOR (95% CI)	*p*-value
Commercial group (Reference)	1.00	-	1.00	-	1.00	-
Mixed group	0.78 (0.48–1.27)	0.319	0.81 (0.50–1.31)	0.384	0.78 (0.46–1.32)	0.348
ICF group	0.22 (0.09–0.54)	< 0.001	0.22 (0.09–0.54)	< 0.001	0.27 (0.11–0.68)	0.005

OR, odds ratio; aOR, adjusted odds ratio; CI, confidence interval.

^1^Model 2: adjusted for baseline characteristics (age, gender, APACHE II score, admission GCS/RASS).

^2^Model 3: further adjusted for clinical interventions (antibiotics, vasoactive drugs, EN duration, route of feeding, infusion rate).

Model fit: the Hosmer-Lemeshow test indicated good goodness-of-fit for the final model (*p* = 0.634).

### 3.4 Gastrointestinal tolerance and metabolic outcomes

Secondary outcomes regarding tolerance and metabolic control are detailed in Table 5. The ICF group exhibited superior gastric tolerance, with markedly lower mean daily gastric residual volumes compared to the Commercial group (27.95 vs. 83.49 mL; *p* < 0.001). Metabolic control was also significantly better, with a drastically lower incidence of hyperglycemia in the ICF group (25.0% vs. 99.6%; *p* < 0.001). Importantly, the improvements in diarrhea and gastric tolerance did not come at the cost of constipation, as the incidence of constipation was comparable between groups (*p* = 0.198).

**Table 5:** Gastrointestinal tolerance and metabolic outcomes.

Variables	Commercial group (*n* = 477)	Mixed group (*n* = 84)	ICF group (*n* = 44)	*p*-value
Gastrointestinal tolerance				
Gastric residual volume (mL/d), median [IQR]	0.0 [0.0–150.0]	0.0 [0.0–0.0]	0.0 [0.0–0.0]	< 0.001
Constipation incidence, *n* (%)	188 (39.4%)	40 (47.6%)	18 (40.9%)	0.369
Duration of constipation (days), mean ± SD	1.82 ± 2.70	2.27 ± 2.65	2.39 ± 3.38	0.198
Metabolic outcome				
Hyperglycemia incidence, *n* (%)	475 (99.6%)	19 (22.6%)	11 (25.0%)	< 0.001
Composite outcome				
Any GI complication,^**1**^ *n* (%)	383 (80.3%)	59 (70.2%)	24 (54.5%)	< 0.001

^1^Composite outcome: defined as the occurrence of any one of the following: diarrhea, constipation, or gastric retention.

In multivariable analyses (Table 6), ICF was independently associated with a significant reduction in gastric residual volume (*β* = −47.25 mL, *p* = 0.029) and a markedly lower risk of hyperglycemia (aOR < 0.001). Overall, patients in the ICF group had 64% lower odds of experiencing any composite gastrointestinal complication (diarrhea, constipation, or gastric retention) compared to the Commercial group (aOR = 0.36, 95% CI: 0.19–0.70; *p* = 0.002).

**Table 6:** Multivariate analysis.

Outcome	Events/total (%) (ICF group)	Adjusted effect measure	aOR (95% CI)	*p*-value
Gastric retention^1^	0/44 (0.0%)^2^	aOR	0.31 (0.12–0.79)	0.014
Hyperglycemia	11/44 (25.0%)	aOR	< 0.001 (< 0.001–0.006)	< 0.001
Composite GI complication	24/44 (54.5%)	aOR	0.37 (0.19–0.71)	0.003

Model adjustment: all models were adjusted for baseline covariates (Age, Gender, APACHE II score, admission GCS/RASS) and clinical interventions (Antibiotics use, vasoactive drug use, duration of EN, infusion rate, route of feeding).

Reference group: the commercial group served as the reference (aOR = 1.00) for all comparisons.

^1^Gastric retention: defined as the presence of gastric residual volume > 100 mL/day or clinician-documented retention.

^2^Statistical note: while the median volume was 0, the logistic regression modeled the probability of having any significant retention event vs. none, demonstrating a robust protective effect.

### 3.5 Sensitivity analyses

Sensitivity analyses confirmed the robustness of our primary findings. Importantly, analyses excluding the Mixed group yielded consistent results, supporting the robustness of the primary comparisons. The association between ICF and reduced diarrhea risk remained statistically significant after excluding the Mixed group (*p* < 0.05). Furthermore, in a propensity score-matched cohort (*n* = 44 per group) that balanced baseline covariates including vasoactive drug use, the ICF group retained its significant advantages in gastrointestinal tolerance and diarrhea reduction (Supplementary Table S1). Subgroup analyses stratified by APACHE II scores showed no significant interaction, indicating consistent benefits across different levels of disease severity.

## 4 Discussion

In this single-center retrospective cohort study, we observed that, under standardized hospital-controlled preparation conditions, ICF was associated with better gastrointestinal tolerance compared with CEN. Specifically, patients receiving ICF experienced a significantly lower incidence of diarrhea (aOR = 0.27, 95% CI 0.11–0.68), a 71% reduction in feeding interruption rate (IRR = 0.29, 95% CI 0.18–0.45), higher mean daily caloric intake, reduced gastric residual volumes, a markedly lower incidence of hyperglycemia, and an approximately 64% reduction in the risk of composite gastrointestinal complications. These findings suggest that ICF may be associated with improved feeding tolerance and metabolic control; however, the retrospective design precludes causal inference. In addition, the relatively small sample size of the ICF group may limit statistical power and the stability of multivariable models. Although sensitivity analyses were performed, the precision of effect estimates may still be affected. However, the possibility of confounding by indication should be considered when interpreting these findings. The lower use of vasoactive drugs and numerically higher admission GCS/RASS values in the ICF group may suggest that some patients receiving ICF were relatively more stable at baseline, which could have exaggerated the apparent benefit of ICF. Conversely, in our clinical practice, ICF was often introduced after poor tolerance to standard commercial formulas or when patients required individualized nutritional adjustment, which may have preferentially selected patients at higher gastrointestinal risk into the ICF group and thus biased the association toward the null. Taken together, the net direction and magnitude of this bias are uncertain. Interpretation of findings related to the Mixed group is inherently complex due to heterogeneous exposure patterns, as patients may have received varying proportions of CEN and ICF during their ICU stay. The relatively strict definition of gastric intolerance (GRV > 100 mL) may have increased sensitivity but also contributed to an overestimation of intolerance and potentially exaggerated between-group differences.

Our findings are broadly consistent with the existing, albeit limited, body of literature examining blenderized or natural-food–based enteral feeding in critically ill populations. Fabiani et al. (12) reported in a cohort of 215 cardiac surgery ICU patients that hospital-prepared blenderized natural enteral feeding administered as bolus feeding was associated with a lower unadjusted probability of diarrhea compared with commercial formulas (log-rank *p* = 0.023), with a trend toward a 42% risk reduction after multivariable Cox adjustment (HR = 0.584, 95% CI 0.335–1.018). Similarly, Schmidt et al. (8), in a small randomized controlled trial involving neurocritical care patients, observed reductions in watery stools and diarrhea frequency with natural food–based tube feeding.

Recent reviews further support this direction of effect. A scoping review by Sforza E et al. (13) summarized multiple observational studies reporting improvements of 30–50% or greater in diarrhea or constipation outcomes with blenderized tube feeding (BTF). A more recent systematic review by Breik et al. (14) concluded that BTF was associated with clinically relevant reductions in diarrhea; however, the certainty of evidence was rated as low, and evidence for other outcomes, such as nutritional adequacy or tube occlusion, was deemed very low. In addition, a meta-analysis by Cara et al. (32) focusing on fiber supplementation rather than BTF per se demonstrated reductions in diarrhea severity (pooled mean difference −2.78) and a 39% reduction in composite gastrointestinal risk. Collectively, these studies, most of which are observational or small-scale trials with substantial heterogeneity, highlight a consistent signal toward improved gastrointestinal tolerance but underscore the lack of high-quality randomized evidence (8, 12–14, 32).

Several mechanisms may plausibly explain the observed advantages of ICF. Compared with commercial formulas, hospital-compounded ICF may have lower osmolarity, retain natural dietary fiber, and preserve a more intact food matrix (8, 9, 11). These characteristics may promote short-chain fatty acid production, enhance colonic water absorption, and reduce mucosal irritation, thereby improving gastrointestinal tolerance (8, 9, 11). At the same time, the literature has highlighted potential risks associated with blenderized tube feeding, including microbial contamination particularly in non-standardized settings feeding tube occlusion, electrolyte disturbances, and variability in nutrient composition (15, 33). In the present study, several procedural safeguards were implemented to mitigate these risks, including preparation in a clean hospital environment, immediate reconstitution, and single-dose dispensing. Nonetheless, microbiological cultures and long-term safety surveillance were not performed, and the possibility of unmeasured safety concerns cannot be excluded.

The findings related to hyperglycemia warrant particularly cautious interpretation. The exceptionally high incidence of hyperglycemia observed in the Commercial group may reflect the use of a relatively broad definition (any blood glucose measurement >10 mmol/L), the severity of physiological stress in critically ill patients, higher use of corticosteroids or vasoactive agents, differences in carbohydrate load across formulas, or variability in local insulin management protocols (29, 34–36). Although the between-group contrast was statistically robust, its clinical relevance is uncertain and may overestimate the metabolic advantages of ICF. Future studies should employ more stringent and clinically meaningful definitions of hyperglycemia such as sustained hyperglycemia or insulin-requiring events and should carefully control for relevant confounders.

From a clinical perspective, CEN formulas used in Chinese ICUs are often imported and associated with relatively high costs (16, 33, 37). In contrast, ICF may offer economic advantages and greater flexibility for individualized nutritional prescriptions, such as low-potassium, high-sodium, or diabetes-adapted formulations, particularly in patients requiring prolonged EN or in resource-limited settings (16, 33, 37). However, current international guidelines (1, 2) continue to prioritize standardized commercial formulas, with blenderized feeding generally considered a secondary option, most commonly in pediatric or chronic tube-fed populations. Although our findings suggest potential benefits of hospital-prepared ICF, the observational nature of the evidence is insufficient to justify changes in routine practice. Widespread implementation would require overcoming substantial barriers, including the availability of trained nutrition teams, standardization of preparation protocols, infection control considerations, and regulatory oversight.

### 4.1 Limitations

This study has several important limitations. First, the single-center retrospective design is inherently susceptible to selection and indication bias. The ICF group was relatively small (*n* = 44) and had a lower prevalence of vasoactive drug use compared with the Commercial group (43.2% vs. 64.6%), raising the possibility of residual confounding despite propensity score matching. The ICF group was relatively small (*n* = 44), which may limit statistical power and the stability of multivariable regression models. In particular, the number of outcome events relative to the number of covariates may not fully meet conventional EPV recommendations, raising the possibility of model overfitting or unstable estimates. Although we conducted sensitivity analyses using reduced-variable models and observed consistent directions of association, these results should be interpreted with caution. Larger prospective studies are needed to confirm the robustness of these findings. Second, multiple secondary outcomes were analyzed without formal adjustment for multiple comparisons, which increases the risk of type I error and warrants cautious interpretation of these findings. Third, the gastric residual volume threshold (>100 mL) used to define intolerance was more stringent than that recommended by ASPEN guidelines (250–500 mL), potentially amplifying between-group differences. Additionally, the definition of gastric intolerance using a GRV threshold >100 mL is more stringent than current guideline recommendations (250–500 mL). This may have led to an overestimation of intolerance events and amplified between-group differences. Therefore, these findings should be interpreted with caution, particularly when comparing with studies using higher GRV thresholds. Fourth, the study did not assess microbiological contamination, feeding tube occlusion, electrolyte or micronutrient abnormalities, long-term nutritional status, or infection-related outcomes. Fifth, the heterogeneity of the Mixed group complicates interpretation. Finally, the use of prokinetic agents, probiotics, and laxatives was not systematically controlled for and may have influenced gastrointestinal outcomes. In addition, standardized nutritional assessment data (e.g., NRS-2002 scores) were not available for inclusion in this retrospective analysis, although such assessments were routinely performed in clinical practice. The absence of these data limits our ability to fully evaluate baseline nutritional comparability between groups and raises the possibility of residual confounding. Furthermore, the decision to initiate ICF was based on clinical judgment, which may introduce confounding by indication, as patients receiving ICF may have differed systematically from those receiving standard commercial formulas. Furthermore, detailed quantitative data on micronutrient composition and dietary fiber content were not consistently available due to the retrospective design and individualized preparation of ICF. As a result, we were unable to formally adjust for nutrient composition differences across groups, which may represent an important source of residual confounding. Additionally, the definition of the Mixed group introduces potential exposure misclassification. Because patients in this group received both CEN and ICF, often in response to changes in clinical condition, the relative proportion and duration of each feeding modality could not be reliably quantified in this retrospective dataset. This may have resulted in non-differential misclassification, potentially attenuating true associations and complicating interpretation. Therefore, results involving the Mixed group should be interpreted with caution. Furthermore, the definition of hyperglycemia as any single blood glucose value >10 mmol/L may have led to overestimation of clinically meaningful events, particularly in the context of frequent ICU monitoring. The lack of data on sustained hyperglycemia, repeated measurements, or insulin treatment limits our ability to distinguish transient from clinically significant hyperglycemia. Sensitivity analyses using stricter definitions were not feasible due to data limitations. Additionally, several clinically important confounders were not included in the multivariable models. In particular, data on corticosteroid use, insulin therapy, and detailed macronutrient composition of enteral formulations were not consistently available in this retrospective dataset. These factors may substantially influence both diarrhea and hyperglycemia and therefore represent potential sources of residual confounding. As a result, the observed associations should be interpreted with caution.

## 5 Conclusion

In summary, this study provides preliminary observational evidence supporting an association between hospital-prepared ICF and improved gastrointestinal tolerance, as well as reduced risks of diarrhea and selected metabolic complications, in critically ill ICU patients. However, the level of evidence remains limited. Future multicenter randomized controlled trials incorporating standardized outcome definitions, long-term follow-up, comprehensive safety monitoring, nutritional equivalence assessments, and cost-effectiveness analyses are needed to establish causality and inform guideline recommendations. Until such evidence is available, the use of ICF should be restricted to centers with specialized expertise and rigorous monitoring protocols.

## Data Availability

The original contributions presented in the study are included in the article/Supplementary material, further inquiries can be directed to the corresponding author/s.
