# Supplementary material for: Risk of gastrointestinal intolerance and complications associated with homemade versus commercial enteral nutrition in critically ill patients: a single-center retrospective cohort study
Source: Front Nutr. 2026 Apr 23;13:1803903. doi: 10.3389/fnut.2026.1803903 (PMC13149064; doi:10.3389/fnut.2026.1803903)
Supplement: Supplementary file 1 [file Table_1.docx]

**Supplementary Table S1**

**Representative commercial and individualized enteral nutrition formulas and their macronutrient and fibre characteristics**

**Part A. Representative commercial enteral nutrition formulas**

| **Formula category** | **Representative product(s)** | **Typical energy density** | **Protein characteristics** | **Carbohydrate characteristics** | **Fat characteristics** | **Dietary fibre information** | **Micronutrient information** |
| --- | --- | --- | --- | --- | --- | --- | --- |
| Standard whole-protein formula (fibre-free) | Nutrison | 1.0 kcal/mL | Whole protein (milk-based) | Standard CHO composition | Standard fat composition | Fibre-free | Nutritionally complete |
| High-energy formula (fibre-free) | Nutrison Energy | 1.5 kcal/mL | Whole protein | Energy-dense CHO | Increased fat contribution | Typically fibre-free | Nutritionally complete |
| Peptide-based (semi-elemental) formula | Nutrison Peptisorb | 1.0 kcal/mL | Peptide-based protein | Easily absorbable CHO | Low fat / MCT-enriched | Fibre-free | Nutritionally complete |
| Diabetes-specific formula (fibre-containing) | Nutrison Advanced Diason | ~1.0 kcal/mL | Soy protein | Low glycaemic index CHO | Modified fat profile | Mixed soluble + insoluble fibre | Nutritionally complete |
| Fibre-enriched formula | Nutrison Multi Fibre / Protein Plus MF | 1.0–1.5 kcal/mL | Whole protein | Standard CHO | Standard fat | Added fibre blend (~10–15 g/L) | Nutritionally complete |
| High-protein formula | Nutrison Protein Plus / Protein Advance | 1.25–1.5 kcal/mL | High protein (≥20% energy) | Standard CHO | Standard fat | ± fibre (variant-dependent) | Nutritionally complete |
| Formula category | Representative product(s) | Typical energy density | Protein characteristics | Carbohydrate characteristics | Fat characteristics | Dietary fibre information | Micronutrient information |

**Part B. Representative individualized compounded formulas (ICF)**

| Formula category | Representative product(s) | Typical energy density | Protein characteristics | Carbohydrate characteristics | Fat characteristics | Dietary fibre information | Micronutrient information |
| --- | --- | --- | --- | --- | --- | --- | --- |
| Individualized compounded formula (overall) | Hospital-prepared ICF | Variable (~1.0 kcal/mL after dilution) | Highly variable (standard to high-protein) | Adjustable | Adjustable | May be fibre-free or fibre-containing | May include added vitamins, minerals, and functional components |
| Low-sodium individualized formula | Hospital-prepared low-sodium EN (powder-based) | Variable (after reconstitution) | ~20.8 g/100 g powder | ~62.5 g/100 g powder | ~8.3 g/100 g powder | ~3.4 g/100 g powder (fibre-containing) | Sodium-restricted; additional components include prebiotics/amino acids |
| Whey protein–enriched formula | Hospital-prepared whey protein EN | Variable (after mixing) | High protein (~80 g/100 g powder) | ~6 g/100 g powder | ~6 g/100 g powder | Not specified on label | Limited micronutrient data available |
| Fibre-containing liquid formula used in ICF practice | Ready-to-use liquid EN | ~101 kcal/100 mL | ~4.18 g/100 mL | ~8.14 g/100 mL | ~5.44 g/100 mL | ~1.44 g/100 mL | Contains multiple vitamins and minerals |
