# Supplementary material for: Risk of gastrointestinal intolerance and complications associated with homemade versus commercial enteral nutrition in critically ill patients: a single-center retrospective cohort study
Source: Front Nutr. 2026 Apr 23;13:1803903. doi: 10.3389/fnut.2026.1803903 (PMC13149064; doi:10.3389/fnut.2026.1803903)
Supplement: Supplementary file 2 [file Table_2.docx]

**Supplementary Table S2**

**Title:** **Comparison of Clinical Outcomes Between Commercial and Homemade Enteral Nutrition Groups After Propensity Score Matching (PSM)**

**(标题：倾向评分匹配后商品组与自制组临床结局的比较)**

| **Variables** | **Commercial Group (n = 44)** | **Homemade Group (n = 44)** | **P-value** | **SMD** |
| --- | --- | --- | --- | --- |
| **Primary Outcome** |  |  |  |  |
| Incidence of Diarrhea, n (%) | 17 (38.6%) | 6 (13.6%) | **0.008** | 0.592 |
| **Secondary Outcomes** |  |  |  |  |
| **Gastrointestinal Tolerance** |  |  |  |  |
| Gastric Residual Volume (mL/d), Mean ± SD | 78.5 ± 120.4 | 27.9 ± 76.9 | **0.024** | 0.498 |
| Constipation Days (d), Mean ± SD | 1.9 ± 2.5 | 2.4 ± 3.4 | 0.452 | 0.165 |
| **Metabolic Outcome** |  |  |  |  |
| Hyperglycemia, n (%) | 43 (97.7%) | 11 (25.0%) | **<0.001** | 2.850 |
| **EN Implementation** |  |  |  |  |
| Incidence of Interruptions, n (%) | 28 (63.6%) | 13 (29.5%) | **0.001** | 0.720 |

**Note:**

- **Matching Variables**: Patients were matched 1:1 using nearest neighbor matching based on Age, Gender, APACHE II score, SOFA score, Admission GCS/RASS, Antibiotics use, and Vasoactive drug use.
- **Data Presentation**: Data are presented as n (%) or Mean ± SD.
- **SMD**: Standardized Mean Difference. SMD > 0.1 indicates imbalance; however, here it reflects the magnitude of difference in *outcomes*.
